# Interactive guidelines: Public communication of data-based research in cities

**DOI:** 10.1371/journal.pone.0228008

**Published:** 2020-01-31

**Authors:** Sergio Trilles, Carlos Granell, Auriol Degbelo, Devanjan Bhattacharya

**Affiliations:** 1 Institute of New Imaging Technologies, Universitat Jaume I, Castellón de la Plana, Spain; 2 Institute of Geography, University of Osnabrück, Neuer Graben, Osnabrück, Germany; 3 CMR Institute of Technology, Bengaluru, India; 4 MagIC, NOVA Information Management School, Universidade Nova de Lisboa, Lisbon, Portugal; Universitat Luzern, SWITZERLAND

## Abstract

Scientific research results are traditionally published as articles in peer-reviewed conference proceedings or journals. These articles often use technical jargon, which precludes the general public from consuming the results achieved. New ways to communicate scientific results are thus necessary to transfer scientific insights to non-experts, and this work proposes the concept of interactive guidelines to fill this gap. A web tool, called Interactive Guidelines Tool, was developed as a proof-of-concept for the idea. It was used in the context of the GEO-C project to communicate research outputs in smart cities scenarios to the public. A comparative analysis between the Interactive Guidelines Tool and related tools helps to highlight the progress it enables beyond the current state of the art. Interactive Guidelines Tool is available as an open-source tool and can be customised/extended by any interested researcher, in the process of making scientific knowledge and insights more accessible and understandable to a broader public.

## 1 Introduction

Today millions of academic papers are published every year without a substantial impact in our society. New mechanisms are needed to transfer scientific knowledge and to mitigate the gap between society and science [1]. The communication of scientific results to society is a significant challenge due to the inherent complexity of scientific findings and the technical and specialised terminology that is used to describe them [2]. Because of that, the vast majority of scientific communications in the form of academic papers and articles hardly reach public audiences, which undoubtedly limits the impact and awareness of scientific discoveries [3]. By available we refer here to the lack of *access* to scientific publications in terms of understanding their implications and impacts in the society. For example, the promotion of open access publishers instead of pay-wall publishers for making available peer-reviewed publications is gaining considerable momentum (e.g., PlosONE and PeerJ), especially by the collective of researchers and research institutions such as universities and public research centers. Making a scientific open access paper is a necessary step to make scientific discovery accessible to anyone, but the spectrum of the society that may benefit from open access policies is in practice limited to communities of specialised researchers and domain experts. A scientific paper in open access, as it is today, implies that a person can digitally access it and read it, but it does not necessarily mean that a person can understand it. Its scientific content is not directed to everyone, but only to a minority. For this reason, there is a growing demand to communicate scientific discoveries *in plain text* to reach a broad audience, changing the traditional style of scientific writing by narrative-driven communication styles through the use of communication channels different from conventional academic journals such as blogs, social networks, and media.

The concept of science communication focuses on the way to communicate scientific findings to the general public [4, 5]. It attempts to reduce the communication gap between researchers, as authors of scientific publications, and society, as the main recipients of scientific discoveries [6]. There are several models to communicate science in bibliography, the dissemination model (often called the deficit model), the dialogue model, and the participation model [7]. These models are not exclusive and can be used together for the communication of science. Hetland et al. [7] argues the importance to increase the dialogue and participation within all three main models.

From the scientific perspective communication itself is not enough, it needs new forms of interaction between society and scientists. Citizen science can be helpful in the process of involving people in new forms of interaction, generating new challenges for scientists and citizens, augmenting scientific knowledge [8].

The strategies to carry out public communication are varied in scope and intensity [9, 10]. Examples are the EU recommendations/policies to push researchers to disseminate their scientific results to general audience [11]. The use of social media metrics or altmetrics [12, 13] is proliferating too. Altmetrics measure the importance of scientific papers based on their aggregated impact in diverse social media sources. In general, the common ground to the previous initiatives and recommendations is that authors are forced to transfer the latest discoveries and scientific knowledge to society as part of the process of doing research [9]. In other words, public communication of science should no longer be a side effect of a research project, but rather an activity intrinsically related to other scientific and management activities of a research project.

Kapon et al. [14] differentiate four different features when it comes to transfer scientific results to citizens, in this case to high school students. These are: *Content features* (what to include/omit to achieve the communication goal), *Knowledge organisation features* (structure elements to show the message), *Analogical approaches* (elements to improve the acquisition of knowledge, analogies, metaphors) and *Stories* (exchange information in a narrative way).

Stories can play an important role in the union between science and society, and translate the abstract and logical scientific knowledge into a concrete narration for non-specialists [2]. In this way, one of the most promising strategies to reach the public is storytelling. Although its convenience is arguably to report scientific results to scientists [15], some works argue that storytelling and narrative approaches to disseminate research can increase understanding, interest, and engagement in science [16–18]. Narratives are intrinsically persuasive and offer tactics for science communicators to persuade audiences that would otherwise be resistant or indifferent to scientific knowledge, although that strategy may also raise ethical considerations. A narrative way to communicate may offer some benefits toward a particular set of communicative goals, but some ethical considerations exist at the intersection of narrative influence and the role of science within society [19].

Data visualisation is another essential tool for scientists to communicate research results in an understandable manner. Over the decades, researchers have used different types of visualisations (e.g., graphs, maps, diagrams) as part of their scientific work, either to validate their experiments, to explore data sets or even to exhibit their results to others. Combined with the narratives, they provide scientists with adequate mechanisms to communicate science effectively to the public.

Hecker et al. [20] enumerates a list of seven tips and helpful practices to communicate citizen science. Some are trivial recommendations. However, the sixth tip puts emphasis on the “Use visualisations and storytelling where possible to achieve people’s understanding.”. This way different types of visualisations such as multimedia elements (video, audio, images, etc.), charts and maps are useful to show and effectively transmit the outcomes of scientific research. Another remark posed by Mazumdar et al. [21] is the use of other channels (e.g., Facebook, Twitter, LinkedIn) in addition to traditional channels such as social media, in order to allow science to reach its audience directly.

Nevertheless, different difficulties or barriers are still present when the receiver is the general public. One of these problems is the incomprehensibility of some scientific and particular concepts, which in many cases become impossible for the general public to be understood without previous knowledge in the area. Another problem is the amount of time that the audience needs to assimilate the final message due to lack of scientific literacy. These two barriers, among others, must be taken into consideration by the transmitter (researcher), and they should be conveniently handled to achieve the goal of public communication of science.

This work addresses the public communication of scientific results by bringing the narrative and visualisation communication styles together in the proposed concept of interactive guidelines to communicate research findings to a broader audience. The term *guideline* is seen as a problem-solution pattern. Problems may be diverse, such as social, mobility, environment, and cultural; solutions may involve a combination of theories, datasets, code, apps, services and any other resource that helps to sort out the current problem. A guideline attempts to communicate a narrative in an understandable manner composed of challenges, benefits, and impacts pertinent to a problem-solution pattern. The qualifier *interactive* underlines the ability of the readers to dynamically explore (to certain degree) the guideline’s narrative through visual and interactive means such as graphs, text and maps. We intentionally avoid static content, like paper-based posters, to let people engage dynamically with the content of the guidelines. In summary, the interactive guidelines can be regarded as a combination of data-driven stories [22], visual storytelling [16, 23], and interactive figures in reproducible research [24, 25], altogether seen under the lens of a narrative approach to communicating scientific findings in an understandable manner to society.

To assess the concept, we designed and developed an Interactive Guidelines Tool (IGT) for the creation, sharing and visualization of interactive guidelines. IGT is part of the Open City Toolkit (OCT), a collection of datasets, tools, services, specifications, and guidelines to deliver services based on open data that are useful for citizens, businesses and governing bodies [26]. The OCT combines technology-driven and citizen-centric strategies to address the lack of integrated and open collections of software components to realize smart cities [27, 28]. The main objective of the IGT is to reveal city stories, i.e., delivering and making visible successful (or not) experiences to city stakeholders to realise an open and transparent city. Therefore, the contributions, and corresponding structure, of this paper are: 1) to define the interactive guideline concept; 2) to describe the design of a conceptual framework and the development of the IGT to create, publish and share interactive guidelines; 3) to test the IGT by communicating the results of the GEO-C research project (a Marie Skłodowska-Curie Actions Innovative Training Network, European Joint Doctorates program funded by the European Commission; http://www.geo-c.eu); and 4) to perform a validation of the IGT using the analysis comparison technique [29] with similar tools in the literature.

## 2 Methods

### 2.1 Interactive guidelines: Need and concept

We explain the concept of interactive guidelines by borrowing an analogy from MIT researcher Cesar Hidalgo about open data sites and supermarkets: “*Imagine shopping in a supermarket where every item is stored in boxes that look the same. Some are filled with cereal, others with apples and others with shampoo. Shopping would be an absolute nightmare!*” [30]. Hidalgo argued that most, if not all, open data sites are organised like arrays of “brown boxes” in supermarkets, i.e., collections of links to public datasets that quite often are published as they were collected. This way, most of these sites look like they are only addressing a small portion of the whole population: those with technical skills (programmers, researchers, etc.) or professionals (e.g., data-driven journalists, civic agents, etc.), i.e., those few specialists who are able to handle and transform datasets to tell stories to the rest of people.

If we do not consider the technological elite, which is the remaining 95% of the population [31], open data sites become challenging to understand (see, e.g., [32, 33]). Returning to Hidalgo’s analogy of the supermarket, imagine a person (citizen) who is asking for “cannelloni” in the food section and the clerk delivers her a bag with all the raw ingredients to cook them themselves. Like most of the open data sites, open data is delivered in the way in which it was collected. Next, that person looks again at the clerk and orders cannelloni “ready to be eaten”, because she does not have time or does not know to cook them. Like most open data sites, open data is not delivered in the way it can best be used and understood. Rather, open datasets are often delivered with no further information on how to process them, manage them, or, even worse, whether they can be useful for citizens at all. In sum, citizens would benefit from “ready-to-consume, easy-to-understand products” rather than raw ingredients like open datasets. Sometimes these products take the form of apps, services, or can also be expressed as interactive guidelines.

Most open data sites do not deliver elaborated stories that emerge from the combination of their contained open datasets. However, often people look for stories (“cannelloni”) that can be readily comprehended (“eaten”).

What we pursue here is the design and creation of guidelines that bring together, behind the scenes, various datasets and other types of resources and transform them into interactive guidelines to make city problems and subsequent actions understandable to citizens regardless the complexity of the details.

The concept of interactive guidelines is envisioned as a way to explore the content and perspectives of research results as an entire, “processed product” instead of looking at the constituted ingredients. Interactive guidelines, as their name suggests, are guidelines (i.e., they walk the user through a narrative), and they are interactive (i.e., designed to provide different outputs depending on the actions of the user). A narrative in this context refers to a problem-solution pattern. The problem is a task that is currently challenging to solve, whereas a solution involves a combination of datasets and software to solve the problem (i.e., improve the current situation). Interaction denotes user interface elements which enable the user to move through different aspects of the narrative. The interaction may involve, for instance, to explore data through through responsive plots or maps. The addition of the impact and benefits of the research into a guideline gives the user an idea of the portability and scalability of the presented solution, i.e., what to reasonably expect when it comes to using the solution in another context [34]. Therefore, interactive guidelines intent to bridge between city users (councils, citizens, companies), between technology and society, and between research and the public communication of science. We summarise here the main features of Interactive Guidelines:

- Show scientific research in a narrative-way (storytelling) using interactive web components (charts, pictures, videos, maps, buttons, …) taking citizens as a reader/consumer.
- Support markdown syntax to write the text and add some formats such as headers, emphasis, lists, images or videos.
- Provide different ways to explore data using charts, tables or maps.
- Offer the ability to execute code (Javascript snippets or P5.js) and see the results automatically in a browser applications.

Similar concepts have been proposed in the past (see Section 4), but mostly covering one of the either defining aspects of interactive guidelines. Exposing content in a narrative manner is well covered in the literature [19], especially in the realm of storytelling and communication research [16–18]. Here, we combine textual narrative and dynamic blocks to shape a pattern-solution narrative. Nevertheless, the combination of static and dynamic content is also not new, as it is rooted in the notion of literate programming [35] or, more recently, as computational essays and notebooks [36]. With respect to interactiveness, much research has paid attention to, for example, how user interfaces can be adapted to changing user situations [37, 38]. The novelty of the notion of interactive guidelines is that it sits in between these previous ideas and works, by bringing together narratives wrapped in the form of literate programming documents, augmented with advanced interactive elements, and all aimed to enhance the public communication of science.

### 2.2 The Interactive Guidelines Tool

#### 2.2.1 Conceptual architecture and underlying technology

**Fig 1:**
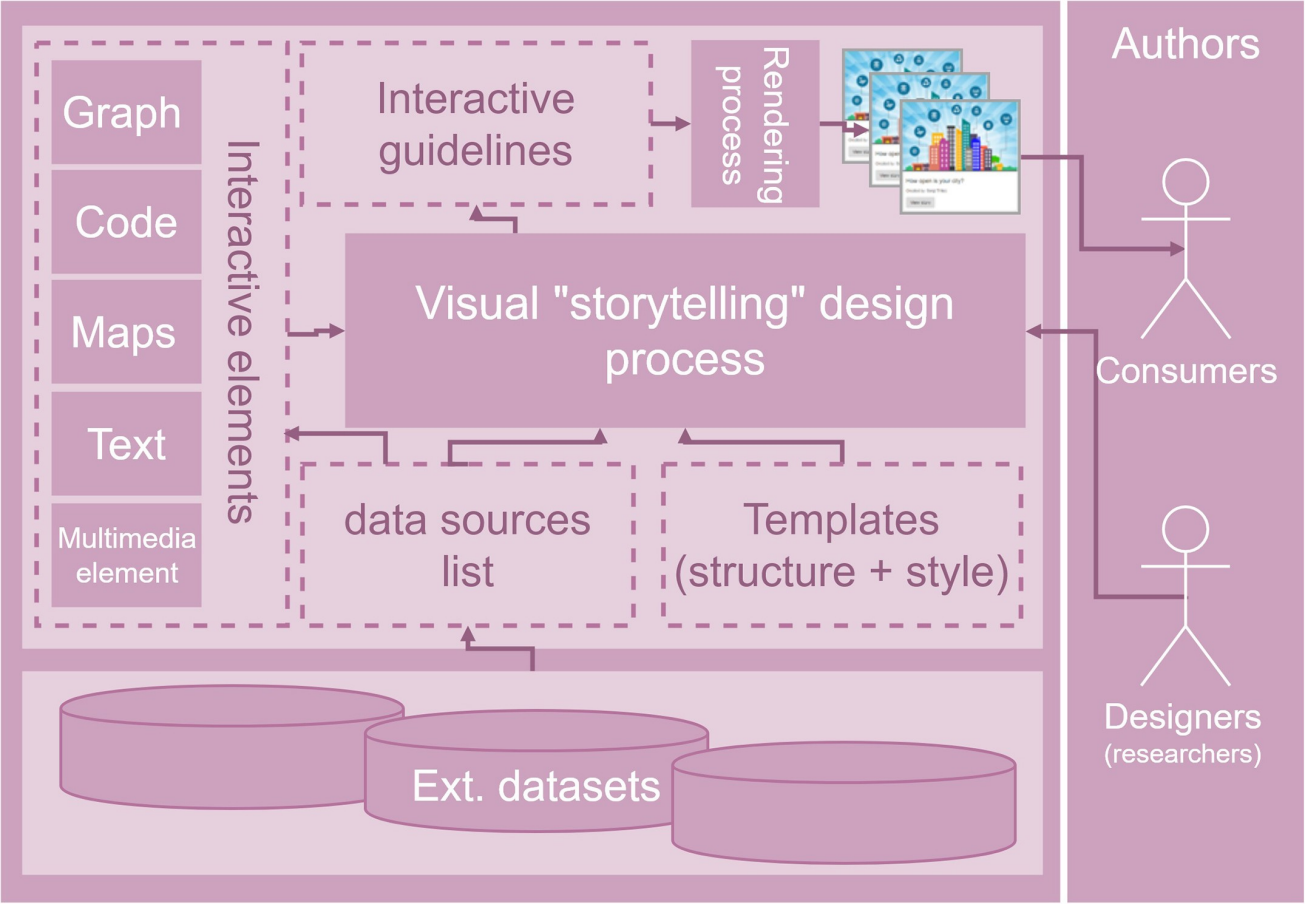
Main components and dataflow of the Interactive Guidelines Tool, which is deployed at http://elcano.init.uji.es/guidelines.

We designed and developed a tool, called Interactive Guidelines Tool [39] (IGT) to create, manage and publish interactive guidelines. The main component of the IGT (Fig 1) is the “Visual Narrative Design” (VND). It allows to create/edit and compose the outline of a guideline. It links all other components such as interactive elements, data sources, and templates. We set up a list of guidelines templates, which come with a predefined structure and a visual style to communicate a narrative. The VND applies a template to ease the creation and rendering of new guidelines. A selection of interactive elements are available through the VND to be added into an interactive guideline (Section 2.2.2). Finally, external data sources can be specified, which are used by the interactive elements to produce data-driven graphs, plots, maps or animations. Two types of end users are proposed: designers and consumers (right side Fig 1). Designers are researchers, scientists or data journalists. They use visual narrative styles to communicate scientific findings. While designers are the primary users of the IGT, city stakeholders (e.g. companies, civic associations, city council, the public) are those who consume the interactive guidelines. Once a guideline is created, the designer renders it and makes it public in a web-based catalogue (Section 3).

From a technological point of view, the IGT is a web-based JavaScript tool based on the Meteor framework. Meteor is a full-stack framework that works both on client and server sides, and combines several JavaScript libraries. Meteor has been specifically designed to create scalable and collaborative applications; it uses a subscription/publication model and provides templates for managing real-time interactions between users in a collaborative way. For the IGT implementation, Meteor’s collaborative features allow designers edit content at the same time that readers can see updates. This adds to the IGT an innovative feature since any changes in the editor mode are automatically synchronised on the client side without updating the web page. In the server side, the IGT is based on Node.js and MongoDB, while the user interface of the client side utilises React.js on top of Meteor.

The IGT can manage (create, edit, or delete) guidelines through users control. It works like a Content Management System (CMS) where users can add new content, in this case, guidelines. The key component is the guideline itself. The definition of a guideline is based on Kajero. It is designed to create single documents, called notebooks. In this way, IGT integrates and extends the Kajero [40] tool to define a guideline and add the functionality to manage these guidelines as detailed in the previous section.

Technically, each guideline is encoded as a Markdown file. Markdown tags specify the sections of a guideline, and keep information about the author, last update, title and the list of data sources used. Each guideline can be exported as a regular markdown file to be edited offline, and may be uploaded again after some edits.

#### 2.2.2 Interactive elements

**Fig 2:**
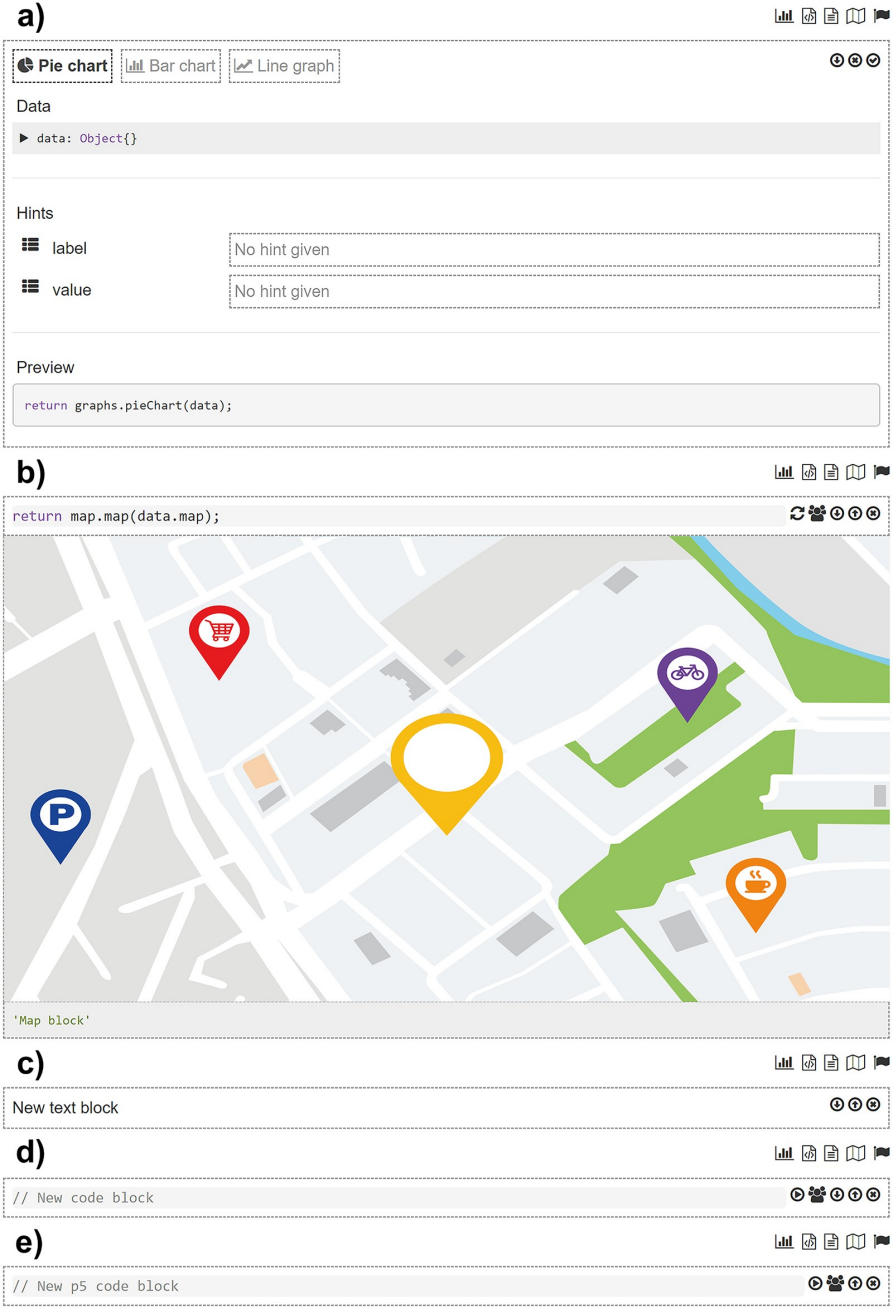
Wizard to configure each interactive block. a) Chart block b) Map block c) Text block d) Code block e) Multimedia element block. This last block is similar but not identical to the original block and is therefore for illustrative purposes only.

The interactive elements or blocks allow designers to add interactivity into a guideline. These elements can be added in any part of a guideline depending on how the designer intends to convey the narrative. The collection of interactive elements, codified as JavaScript snippets, are grouped in these five categories:

- **Charts**. Designers employ this block to visualise defined datasets as a chart. The chart block uses internally a D3-based reusable chart library called Jutsu to create and render charts. Several types of charts are supported such as pie charts, bar charts or line charts. Each chart type is a configurable block (Fig 2(a)) in which designers establish the data source, which may be external datasets or a HTTP query that returns data, and can add other settings such as title axes.
- **Text**. It is the most basic block to add text and other multimedia elements such as videos and images to the guideline. The text block (Fig 2(c)) supports Markdown’ common features such as headers, lists, links and so on. It also visualise images and videos using the corresponding Markdown tags.
- **Code**. This block is able to run JavaScript code chunks entered by the designer (Fig 2(d)). Custom code can be used for example to access external services, or to initialise instances of variables based on data typed by consumers at reading time.
- **Multimedia elements**. This block offers advanced features for producing any kind of interactive animations. This block brings the full potential of the processing language (p5.js) to the designers. It is a powerful animation library to create interactive art, games, data visualisations and animations. As p5.js is written in JavaScript, it works on the web and can use the existing web features and HTML5 APIs such as sound, video, geolocation and webcam. This block does not need any parameter, only the P5 code itself that the designer types directly in the block (Fig 2(e)).
- **Maps**. This blocks visualises geographic data on a map. Again, the source of the geographic data must be specified in the map block (Fig 2(b)), in this case restricted to the GeoJSON-encoded data. The map block uses the Leaflet library to render the final map (Fig 2(f)).

## 3 Results

In this section we show how consumers and designers can interact with the IGT to create and manage interactive guidelines. Examples of interactive guidelines and the evaluation of the IGT are described in the next section 4.

**Fig 3:**
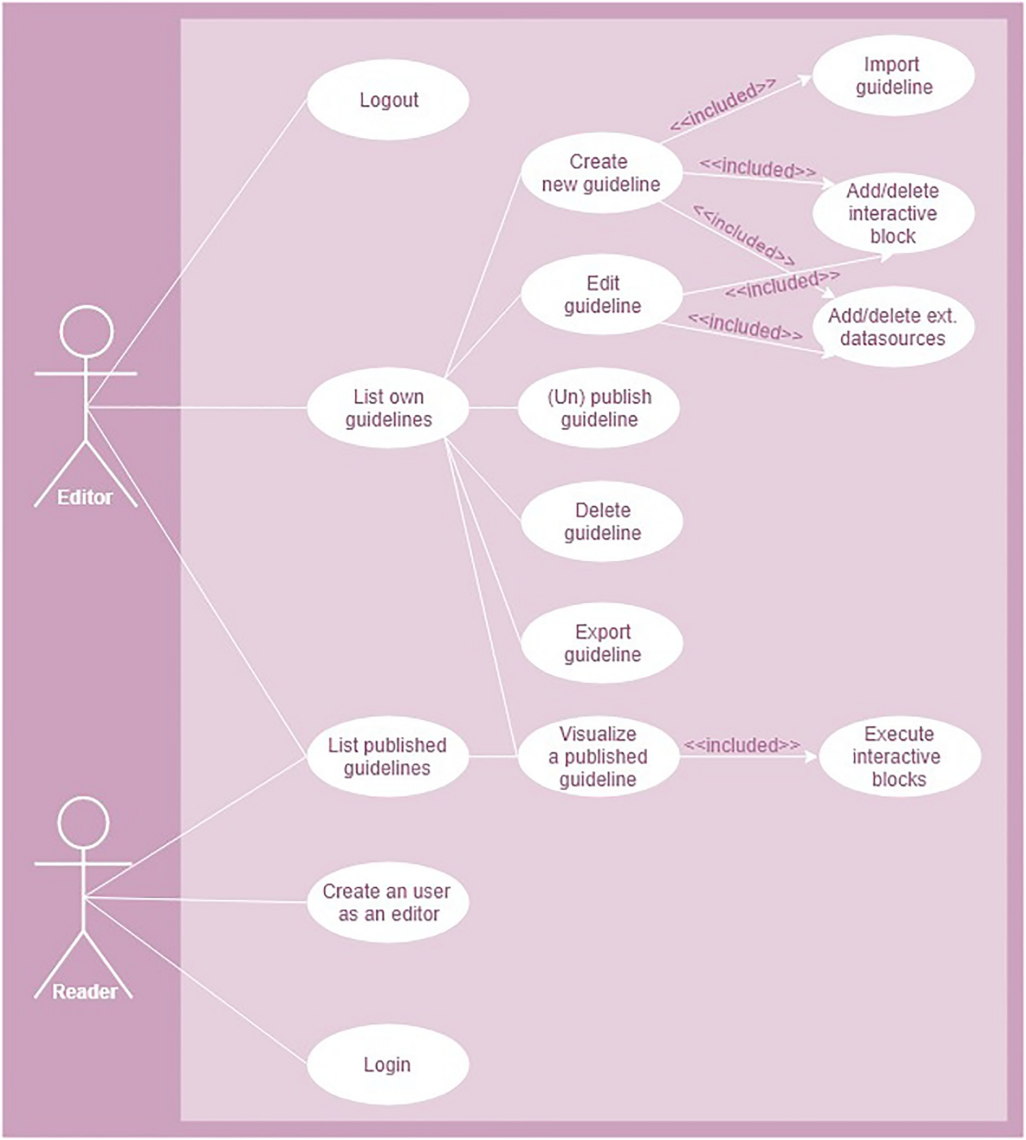
Allowed actions of the two interaction roles (readers and editors) with the IGT.

Consumers and designers play the role of readers and designers, respectively (Fig 3). A reader can access to the list of public guidelines, visualise/interact with them and execute blocks with the possibility to execute option. Like readers, an editor can access, visualise and interact with their own guidelines and others created by other editors. Unlike readers, editors have writing permission over their own guidelines through the following actions: create, edit, publish/unpublish, delete and export. In edition mode, an editor can create a guideline, either using a template or by importing an existing guideline, add/remove interactive blocks and external data sources. An editor can also export guidelines, for off-line work; publish a guideline, make it openly available through the public catalogue; or unpublish it, to make it private.

**Fig 4:**
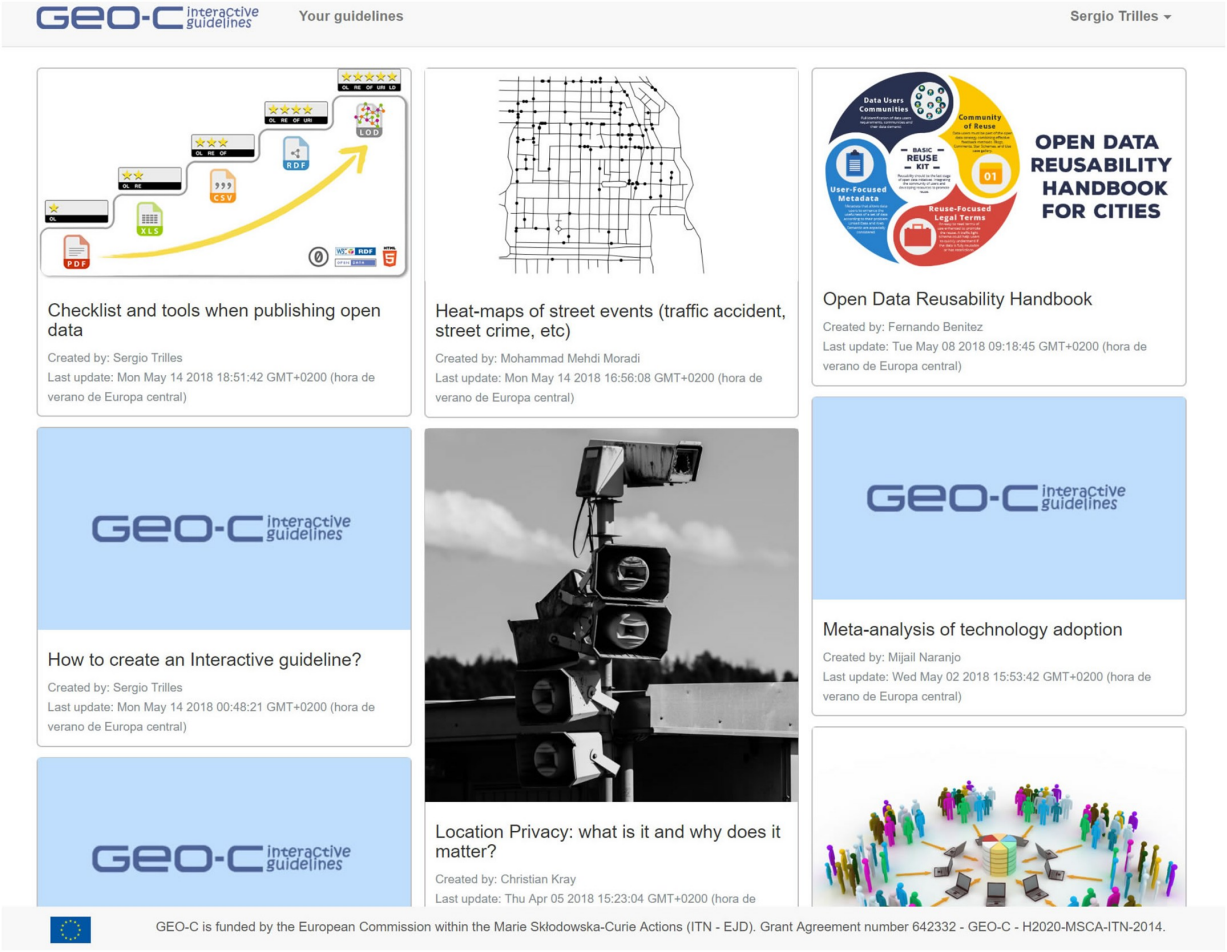
Card-based view of the catalogue of published interactive guidelines, http://elcano.init.uji.es/guidelines.

**Fig 5:**
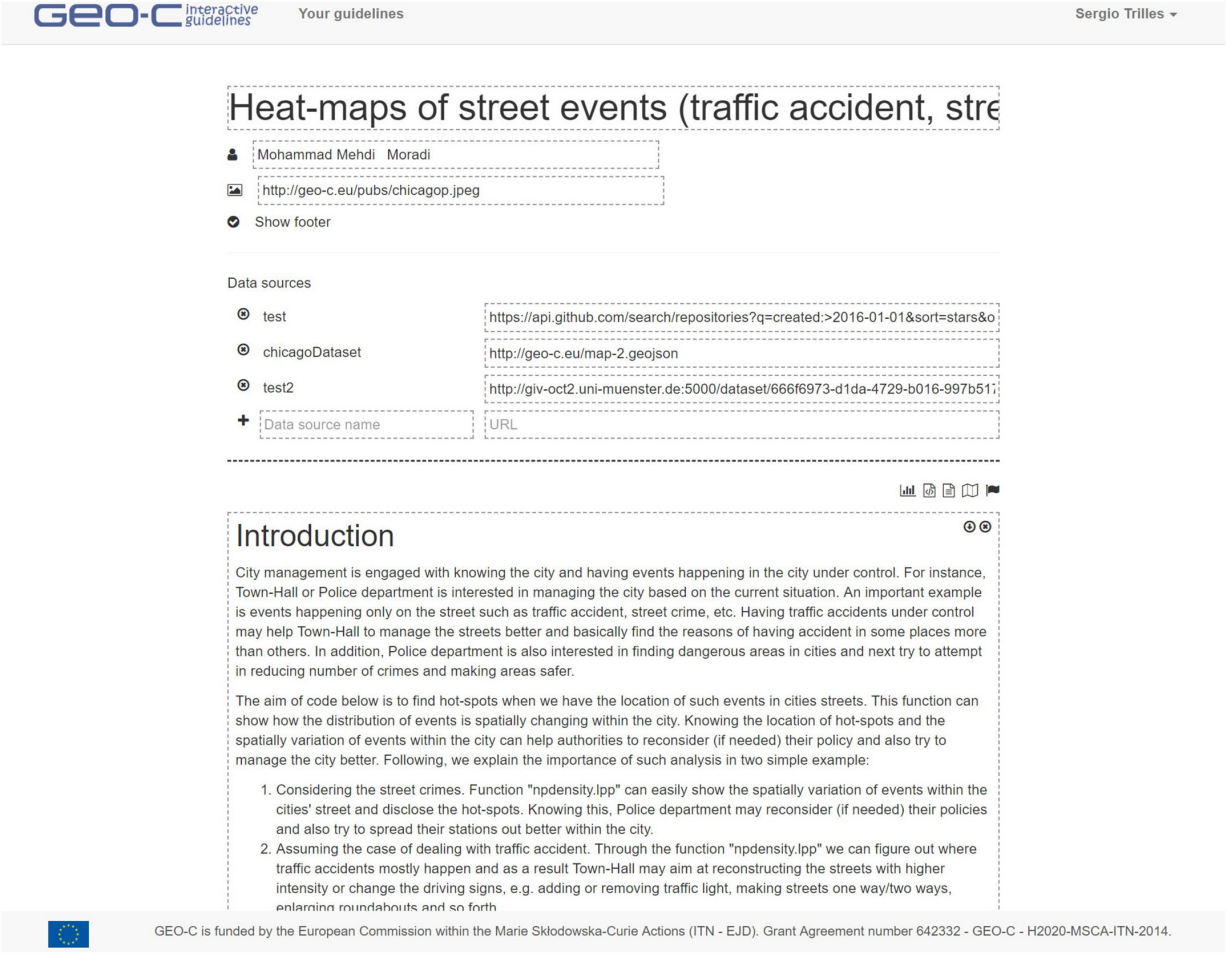
In editor mode, a designer can edit any aspect of an interactive guideline.

The entry point to the published interactive guidelines is the catalogue (Fig 4). The title, author, update date, and a featured image of a guideline appear on each card. When a card is selected, a new browser window opens to show the content of the corresponding guideline. For editors to create a new guideline, some bits of information such as the title and the template selected (or existing guideline) are required to initialise a bare interactive guideline, which is opened in editor mode (Fig 5). At this point, the designer can add a data source, interactive elements, and text shape, and the narrative of the guideline.

A guideline is organised in three parts. The top part contains descriptors such as title, featured image and author name, used in the card-based view of the catalogue. The next part refers to the specification of data sources, which are the input data for the interactive elements. The last part is a series of blocks; the designer adds as many diverse blocks as required to communicate the narrative of the interactive guideline. Once done with the edition, the designer can publish it in the catalogue for public access, or export it as a local file for sharing or off-line work. The exported file contains all of the guideline parts encoded in Markdown format along with the metadata fields. The contained resources (images, data sets, etc.) are conveniently saved using absolute paths so that the complete guideline can be imported back into the IGT without loss of information.

**Fig 6:**
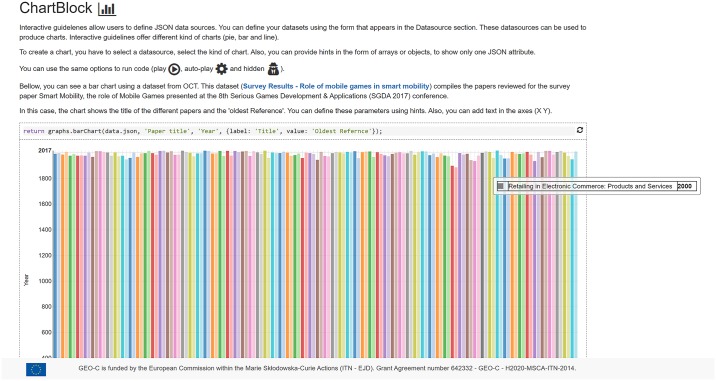
An example of runnable interactive block in the reader mode.

In the reader mode, a reader can only execute and visualise *runnable* blocks of the guideline being visualised. All but the Text blocks are runnable. Each of these blocks has two execution options (Fig 6): a block can be executed by clicking the play button or automatically when the guideline is loaded. In addition, the source code of a block can be set to ‘hidden’, which means that the result of the execution will be visible, while the source code of that block will remain hidden to the reader.

## 4 Evaluation

### 4.1 The GEO-C Open City Toolkit

We take the GEO-C project as a primary use case to assess the interactive guidelines, as the IGT was developed in the context of that project. Because of its particular organisation, 15 doctoral researchers conducted their research in varied but complementary topics, the results of the project can be regarded as the individual research outputs derived from each doctoral project. Despite this diversity, the research topics fell into a few research themes [27]: empowering citizens (*R1*), analytical methods and tools (*R2*), and citizen-centric services (*R3*). *R1* includes deep participation (i.e., work with the community, and not just for the community) and data-literate citizenry (i.e., democratise data literacy skills). *R2* includes pairing quantitative and qualitative data (i.e., provide analytical methods that are able to cope with both types of data) as well as the adoption of open standards (i.e., provide open standards for the access and use of city data). *R3* includes personal services (i.e., provide customised services) and persuasive interfaces (i.e., create new types of user interfaces which present information in such a way that citizens are persuaded to change their behaviour and take actions accordingly).

**Fig 7:**
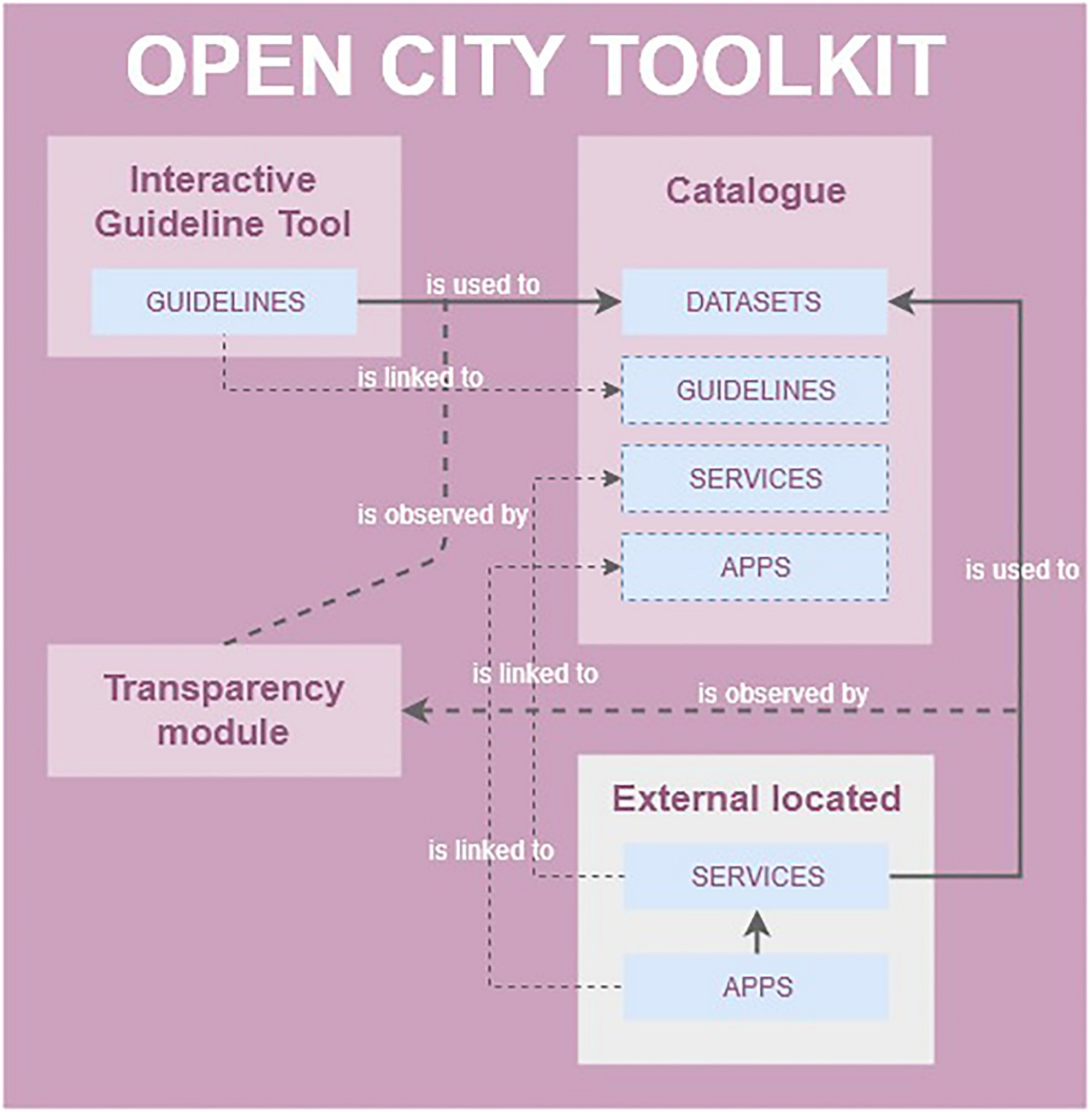
Relationships between the OCT components.

All doctoral students registered their individual research outputs in the Open City Toolkit (OCT) [27]. The OCT was conceived of as a collection of datasets, guidelines, services and apps produced during the GEO-C project to deliver city services based on open data that are useful for citizens, businesses and governing bodies. The OCT is relevant here because interactive guidelines were one type of research outputs within the project. The OCT is composed of three modules (Fig 7), i) the OCT Catalogue; ii) the Transparency module; and iii) the IGT. The OCT Catalogue is a centralised access and storage point to discover and access the research contributions of the doctoral projects. The Transparency module tracks the re-use of datasets in the OCT catalogue by the registration of applications and services that utilised them [41]. Finally, the IGT is a means to communicate and transfer the scientific knowledge acquired during the project to city stakeholders (consumers).

The role of the IGT within the OCT, and thereby in the entire project, was to enable citizen-centric narratives of the project results (concepts, technology, tools, and data).

Technically (Fig 7), the IGT relies on the datasets registered in the catalogue, which are in turn used by the interactive elements of a guideline to generate data visualisations, maps, and animations. This way, research outputs as datasets are directly integrated into the narrative of an interactive guideline. Once a new version of a dataset is loaded into the catalogue, the linked guideline and all the interactive elements that depend on that dataset are automatically updated accordingly. For example, a graph element that produces a bar chart takes the new version of the dataset transparently to the designer. The second relation between the IGT and the OCT catalogue is that all generated guidelines are also registered in the main OCT catalogue, treating them as a first-level research output (see http://giv-oct.uni-muenster.de:5000/group/guidelines).

At the moment of this writing, there are ten guidelines; some of them are like tutorials to designers (e.g., doctoral students) to use and combine interactive elements and data sources into a guideline. Others communicate narratives of scientific results, as the following ones:

- *Location Privacy: what is it and why does it matter? (* https://elcano.init.uji.es/documents/zdpNx7gos2CikSGFg*)*. It communicates the importance and implications of sharing one’s location using smartphones. It uses text blocks to show figures, tables and text. Anchors are also included to guide the content through the guideline.
- *Open Data Reusability Handbook (* https://elcano.init.uji.es/documents/qaBFbFxqPB2tQrf97*)*. It shows a set of recommendations and technical actions to improve the reusability of open data. It is especially designed for local data authorities to capture users’ data demands and requirements following a bottom-up approach. It also covers reusability challenges and barriers that prevent users from taking full advantage of open geographic data. It uses text blocks to show figures, tables, and text, and anchors to navigate the content of the guideline.
- *Citizen Participation And E-Governance Paradigms For Smarter Cities (* https://elcano.init.uji.es/documents/SciNxfiDp2CDJhL33*)*. It explains a step by step procedure for citizens to be actively involved in e-governance processes in their area. It provides data-driven visualisations as examples of priority to citizens.

### 4.2 A metrics analysis against similar works

This section performs a validation of the IGT following the analysis comparison technique [29]. This method is based on making a comparison between existing solutions in similar use cases to the IGT. This evaluation technique defines a set of metrics that are used to describe and compare each of the solutions included in the study. The final objective is to position the IGT tool against other similar literature approaches and show similarities and difference between them.

The inclusion criterion for a study or tool was that it proposes a narrative way to disseminate or communicate research results using guidelines or tutorials. We found a few eligible studies, which are summarised next. To find the eligible documents, we used Google Scholar and the search terms “communication”, “storytelling”, “interactive” and “science” to obtain resulting list of document shown below.

- Grainger et al. [42] designed a framework to enhance the communication and application of scientific information focused on professional contexts. The paper reveals than effective visualisations can improve the dissemination and knowledge of science within environmental decision-making.
- Jun et al. [43] investigated barriers that prevent researchers from communicating science effectively in online experiments. The authors then present a web-based tool that supports researchers in providing their participants with science communication pages.
- Gay and Li [44] explored Web accessibility checkers that often produce different results in the analysis of the same web content. In this context, the AChecker was developed to make accessibility analysis transparent so that users can understand exactly how accessibility is being assessed.
- Groshans et al. [45] uses ESRI Story Map to communicate research in treating climate as a soil forming factor. This kind of digital maps combine data, locations, and people to conveying information and exemplary user experience.
- Porteous et al. [46] have developed a virtual environment using Unity3D engine to demonstrate interactive narrative featuring based on a pre-existing children’s story which allows for the generation of variants of the original story that can be “told” via visualization in the 3D world. An user interface allows experimenters to insert and order cues and specific events while the narrative generation techniques ensure these requests are effected in a consistent fashion. The work reports the results of a field experiment with children (age 9-10) that demonstrates the potential for the use of virtual narratives in story understanding experiments.
- Cavazza et al. [47] have shown a proof-of-concept prototype exploring the concepts of serious games and interactive narratives technologies to achieve clinical guidelines with a real-time and interactive narrative, which provides a principled simulation (using Unreal Engine) of the situations faced by patients, which preserves causal and deontic constraints.

To compare the above studies with the IGT, we have defined a set of metrics, which are described below. Table 1 summarises the comparative analysis between the reviewed studies and the IGT.

- *Approach*: Indicates conceptual architecture. Answer scale: approach description.
- *Interactivity*: Whether or not a work supports interactivity. Answer scale: Yes/No.
- *Storytelling*: Whether or not a work follows a narrative/storytelling approach. Answer scale: Yes/No.
- *Data*: Whether or not a work can utilise published datasets to generate visualisations. Answer scale: Yes/No.
- *Visualisation types*: Whether or not a work supports different types of visualisations such as maps and charts. Answer scale: Yes/No.
- *Multimedia elements*: Whether or not a work supports different types of multimedia elements, such as 3D, sound, camera, geolocation and user inputs. Answer scale: Yes/No.
- *Social media sharing*: Whether or not a work supports the possibility to share the guideline using social media networks such as: Facebook, Twitter, LinkedIn and so on. Answer scale: Yes/No.

**Table 1:** Comparative analysis between eligible studies and the IGT (bottom row).

References	Approach	Interactivity	Narrative or Storytelling	Data	Visualisation types		Multimedia elements					Social media sharing
					Map	Charts	3D	Sound	Cam.	Geo-locat.	User input	
[42]	Conceptual	✔	✔	✔	✔	✔	✔	✘	✘	✘	✘	✘
[43]	No-functional	✘	✘	✘	✘	✔	✘	✘	✘	✘	✘	✘
[44]	WebApp	✔	✘	✘	✘	✘	✘	✘	✘	✘	✘	✘
[45]	WebApp	✔	✔	✔	✔	✔	✔	✘	✘	✘	✘	✘
[46]	3d simulation	✔	✔	✘	✘	✘	✔	✔	✘	✘	✔	✘
[47]	3d simulation	✔	✔	✘	✘	✘	✔	✔	✘	✘	✔	✘
IGT	WebApp	✔	✔	✔	✔	✔	✔	✔	✔	✔	✔	✔

## 5 Discussion

This work presents the concept of Interactive Guidelines to facilitate the communication of scientific results to city stakeholders in particular and society in general. An interactive guideline combines different approaches such as storytelling, narrative styles, and interactive visualisation elements, aimed at describing easily and understandably research outcomes.

To facilitate the creation and management of interactive guidelines, the web-based Interactive Guidelines Tool has been developed in the context of the GEO-C project. Employing the IGT, designers can create their interactive guidelines by combining five different interactive elements or blocks. These elements exploit remote datasets to create data-driven, interactive visualisations and animations in an understandable way. An assessment has been done base on a case study, the interactive guidelines developed on the GEO-C project, together with a comparative analysis with similar studies. The exhaustive search led only to four studies, which indicates how innovative the concept of interactive guidelines and the IGT are, although this field is still in its infancy.

The concept of interactive guidelines does not specifically target technologically savvy people such as open data advocates, researchers, and developers. The interactive guidelines aim to inform people about problems that matter in their cities, making them understandable, and presenting potential solutions. Interactive guidelines, when designed as effective visual narratives, try raise awareness about some urban issues (or any other research topic), even to the point to persuade and frame the thinking of their citizens using storytelling and multimedia elements. In this sense, interactive guidelines could have an educational footprint in the long run. Studies on science communications (e.g., [10]) have pointed out the benefits of having practical, operable, and functional tools for communicating science to the public. To date, there has not been functionally complete tools reported in the literature. Main recommendations from the literature suggest the support of some features such as types of visualisations, interactive elements, data-based visualisations, user interactions, and so on [21]. The IGT presents progress along these lines though much remains to be done.

Immediate directions for future work include further extensions of the tool, such as new interactive blocks, notes/comments from readers (citizens) or datasets metrics provided by the OCT transparency tool. After the performed scientific validation, usability studies and interviews are also necessary to gather feedback towards an improved usability and adoption.
